# Bark Extract of the Amazonian Tree *Endopleura uchi* (Humiriaceae) Extends Lifespan and Enhances Stress Resistance in *Caenorhabditis elegans*

**DOI:** 10.3390/molecules24050915

**Published:** 2019-03-06

**Authors:** Herbenya Peixoto, Mariana Roxo, Emerson Silva, Karla Valente, Markus Braun, Xiaojuan Wang, Michael Wink

**Affiliations:** 1 Institute of Pharmacy and Molecular Biotechnology, Heidelberg University, INF 364, D-69120 Heidelberg, Germany; hspeixoto1@gmail.com (H.P.); marianaroxocorreia@gmail.com (M.R.); markus.braun@gmx.ch (M.B.); wxjsz@hotmail.com (X.W.); 2 Faculty of Pharmaceutical Science, Federal University of Amazonas (UFAM), 6200 General Rodrigo, Manaus 69077-000, Brazil; eslima75@gmail.com (E.S.); karlaacussena@gmail.com (K.V.)

**Keywords:** *Caenorhabditis elegans*, antioxidants, bergenin, stress resistance, lifespan, Huntington, uxi, *Endopleura uchi*

## Abstract

*Endopleura uchi* (Huber) Cuatrec (Humiriaceae), known as uxi or uxi-amarelo in Brazil, is an endemic tree of the Amazon forest. In traditional medicine, its stem bark is used to treat a variety of health disorders, including cancer, diabetes, arthritis, uterine inflammation, and gynecological infections. According to HPLC analysis, the main constituent of the bark extract is the polyphenol bergenin. In the current study, we demonstrate by in vitro and in vivo experiments the antioxidant potential of a water extract from the stem bark of *E. uchi*. When tested in the model organism *Caenorhabditis elegans,* the extract enhanced stress resistance via the DAF-16/FOXO pathway. Additionally, the extract promoted an increase in the lifespan of the worms independent from caloric restriction. It also attenuated the age-related muscle function decline and formation of polyQ40 plaques, as a model for Huntington’s disease. Thus, these data support anti-aging and anti-oxidant properties of *E. uchi*, which has not yet been described. More studies are needed to assess the real benefits of *E. uchi* bark for human health and its toxicological profile.

## 1. Introduction

*Endopleura uchi* (Huber) Cuatrec, popularly known in Brazil as uxi or uxi-amarelo, is an endemic tree found throughout the entire Brazilian part of the Amazon basin [1]. The species belongs to the family Humiriaceae and is the only member of its genus. This valuable tree is locally used for its wood, bark, fruit, and seeds [2].

Traditional medicinal applications of the stem bark of *E. uchi* include the treatment and prevention of cancer, diabetes, high cholesterol, arthritis, diarrhea, and genitourinary disorders, especially uterine inflammations and infections [3]. A recent ethnobotanical survey has reported a high demand for uxi bark in regional markets due to its popular therapeutic claims [4,5,6,7]. However, few studies have investigated the bioactivities of *E. uchi*.

Silva and Teixeira [8] reported the in vitro antioxidant and antibacterial activity of the bark, as well as inhibition of cholinesterase (AChE, BuChE) and α-glucosidase. The authors associated the inhibition of α-glucosidase with the traditional use of the bark to treat diabetes. Additionally, no cytotoxic effect was observed when tested in human colorectal adenocarcinoma cells (Caco-2). When tested in HeLa cells, a polysaccharide fraction of *E. uchi* barks significantly reduced proliferation and cell viability [9]. Sá et al. [10] demonstrated that the subchronic administration of *E. uchi* bark extract has no toxic effects on male and female Wistar rats. Politi et al. [11] also assessed the safety profile of *E. uchi* bark and reported the absence of oral acute toxicity.

Previous phytochemical investigations of *E. uchi* bark have revealed the presence of tannins, terpenoids (saponins and steroids), and coumarins [12,13,14]. The isocumeric secondary metabolite bergenin has been reported by several researchers as the major compound in *E. uchi* bark [8,15,16,17].

In the current study, we investigated a water extract from the stem bark of *E. uchi* regarding its potential antioxidant and anti-aging properties using the nematode *Caenorhabditis elegans* as an experimental model, which is widely used in this context.

## 2. Material and Methods

### 2.1. Plant Material and Extract

*Endopleura uchi* extract (EU) was obtained from stem bark purchased from a local trader in Manaus-AM (Brazil). The bark material was weighed, milled, and exhaustively extracted with distilled water (5 × 1 L) at room temperature during an overall extraction period of 5 days. Using a rotary evaporator, the water extract was concentrated at low pressure at 40 °C, frozen at −80 °C, and finally lyophilized to obtain a fine dried powder. The plant material used in this study is deposited in the sample collection of IPMB (Institut für Pharmazie und Molekulare Biotechnologie, Heidelberg, Germany) under the accession number IPMB P8636.

### 2.2. Antioxidant Activity

In a 96-well microplate, 100 μL of sample were added to 100 μL of 200 µM DPPH. After 30 min, the absorbance was measured in a microplate reader (Tecan Trading AG, Männedorf, Switzerland) at 517 nm [18]. All measurements were performed in triplicate. The EC_50_ is presented in µg/mL.

### 2.3. Total Phenolic Content

In a 96-well microplate, 20 µL of sample were added to 100 µL of Folin-Ciocalteu reagent; after 5 min, 80 µL of sodium carbonate (7.5% solution) were added to the wells. The reaction ran for 2 h protected from the light and at room temperature; the absorbance was measured at 750 nm. The assay was carried out in triplicate and repeated three times. The phenolic content is expressed as gallic acid equivalents (GAE/g of sample).

### 2.4. Chemical Characterization and Quantification of Bergenin

Bergenin content of the uchi extract was determined by high performance liquid chromatography (HPLC) in a Shimadzu Proeminence Chromatograph with a UV-Vis detector SPD-10A. The method used was adapted from Tacon and Nunomura [17,19]. The chromatography was run in gradient mode with methanol: formic acid 0.1% as the mobile phase A, and aqueous formic acid 0.1% as the mobile phase B. The column C-18 SphereClone 5 µ ODS (150 × 4.60 mm and particle size 5 µm) and the detector was set to wavelength of 272 nm. The flow rate of the mobile phase was 0.8 mL/min. The calibration curve was constructed using bergenin (Sigma, St Louis, MO, USA), ranging from 0.04 to 1.5 mg/mL, obtaining a linear correlation coefficient of 0.9995.

### 2.5. C. elegans Strains and Maintenance

The worms were cultivated on NGM plates inoculated with living *E. coli* OP50 as food source and incubated at 20 °C, except when mentioned. For the current work we used the strains N2 (wt), CF1038 (daf-16(mu86)), GR1307 (daf-16(mgDf50)), CF1553 (muIs84 [(pAD76) sod-3p::GFP + rol-6]), AM141 (rmIs133[P(unc-54)Q40::YFP]), TJ375 (gpIs1[hsp-16-2::GFP]), and BA17 [fem-1(hc17) IV)].

Age synchronous cultures were obtained by treating the adult hermaphrodites with a lysis solution (5 M NaOH and 5% NaOCl) for 5 min and separating the eggs from the debris by density gradient centrifugation using 60% sucrose solution [20]. The collected eggs were allowed to hatch in M9 buffer [21].

### 2.6. Survival Assay under Oxidative Stress

For this assay, age synchronized L1 larvae (N2, CF1038, and GR1307 strains) were grown in *S*-medium. The larvae were sorted into groups of 75 individuals and treated with the extract for 48 h. Subsequently, 80 µM of the pro-oxidant juglone (5-hydroxy-1,4-naphthalenedione) were added to each group and 24 h later the number of live and dead worms were scored. We considered a worm to be dead when it did not respond to a gentle touch with a platinum wire [20]. The assay was carried out in triplicate and is presented as mean ± SEM compared by one-way ANOVA followed by Bonferroni (*post-hoc*).

### 2.7. Intracellular ROS Accumulation

For this assay, we used age synchronized L1 larvae (N2 strain) grown in *S*-medium. The larvae were sorted into groups and treated with the extract for 48 h. Subsequently, the ROS-sensitive fluorescent dye H2DCFDA (50 µM) was added to each group. The staining took 1 h, protected from the light, at 20 °C; afterwards the worms were mounted onto a glass slide and paralyzed with a drop of 10 mM sodium azide. Using a BIOREVO BZ-9000 fluorescence microscope (Keyence Deutschland GmbH, Neu-Isenburg, Germany), live images of at least 30 worms per group were captured with an excitation filter set to 480/20 nm and emission filter set to 510/38 nm [20]. The relative fluorescence was determined densitometrically using the software Image J (National Institute of Health, Bethesda, Bethesda, MD, USA). The results are presented as mean fluorescence intensity (mean ± SEM) and compared by one-way ANOVA followed by Bonferroni (*post-hoc*). The assay was repeated three times.

### 2.8. Quantification of Gene Expression Using GFP Reporter

For this assay, we used age-synchronized worms grown in S-medium. To evaluate *sod-3*:GFP expression, L1 larvae (CF1553 strain) were treated with the extract for 48 h and analyzed under a fluorescence microscope, as described above. To evaluate *hsp-16.2*::GFP expression, L4 larvae (TJ375 strain) were treated with the extract for 48 h and subsequently exposed to 20 µM juglone; 24 h later, the worms were analyzed by fluorescence microcopy, as described. The assays were repeated three times and the results are presented as fluorescence intensity (mean ± SEM) compared by one-way ANOVA followed by Bonferroni (*post-hoc*).

### 2.9. Longevity Assay

For this assay, we used age-synchronized worms at day 1 of adulthood (BA17 strain) grown in S-medium. The adults were treated with the extract, except the control group. Throughout the entire period of observation, the worms were incubated at 25 °C and transferred every second day to fresh medium supplemented with extract following their treatment groups. Dead worms were scored during the transfer and removed from the assay. Worms exhibiting extruded gonads or internally hatched progeny were scored as censored and removed from the assay. We considered a worm to be dead when it did not respond to a gentle touch with a platinum wire [22]. The assay was repeated three times and the results are presented as percentage of survival. The statistical significance was determined by Log-rank (Mantel-Cox) tests followed by Gehan-Breslow-Wilcoxon Test.

### 2.10. Quantification of PolyQ40::GFP Aggregate Formation

For this assay, we used age synchronized L1 larvae (AM141 strain) grown in *S*-medium. This strain serves as a model for Huntington disease. The larvae were treated with the extract for 48 h and subsequently submitted to fluorescence microscopy, as described above [22]. The results are presented as number of PolyQ40::GFP aggregates (mean ± SEM) and compared by one-way ANOVA followed by Bonferroni (*post-hoc*).

### 2.11. Pharyngeal Pumping Rate

For this assay, we used age synchronized worms (N2 strain) grown on NGM agar plates. The adult worms were daily transferred to fresh plates, supplemented following their treatment groups, throughout the entire reproductive period. At day 5 and day 10 of adulthood, the worms were observed under a stereomicroscope for 1 min to score the pumping activity of the pharynx, which can serve as a measure for muscle impairment during aging [23]. The results are presented as pumps/min (mean ± SEM) and compared by two-way ANOVA followed by Bonferroni (*post-hoc*).

### 2.12. Body Length

If *C. elegans* is kept under dietary restriction (DR), its body length will decrease. To assess the body length, age synchronized L4 larvae (N2 strain) were treated with the extract for 24 h; subsequently, the worms were mounted onto a glass slide and submitted to bright field microscopy. Live images were taken from at least 30 worms per group; the length was measured from head to tail using the software ImageJ (version 1.48, National Institute of Health, Bethesda, MD, USA). The results are presented as body length in µm (mean ± SEM) and compared by one-way ANOVA followed by Bonferroni (*post-hoc*).

### 2.13. Antimicrobial Activity

Susceptibility of *Escherichia coli* strain OP50 to the extracts was assessed by means of well diffusion test according to CLSI (2014) with slight modifications [24]. Briefly, bacteria were grown on Müller-Hinton agar (MHA) and the cell suspension was adjusted to 0.5 McFarland standard. Wells with 6 mm in diameter were punched out and loaded with 70 µL of 10 mg/mL sample dissolved in sterile water. Ampicillin and ciprofloxacin (256 µg/mL) were used as positive controls. Diameters of the zones of inhibition (ZI) were assessed 24 h after incubation at 35 °C. The assay was repeated three times. The bacteria were purchased from the Caenorhabditis Genetics Center (CGC, University of Minnesota, Minneapolis, MN, USA).

## 3. Results

### 3.1. Antioxidant Activity in Vitro and Chemical Characterization of the Bark Extract

**Figure 1:**
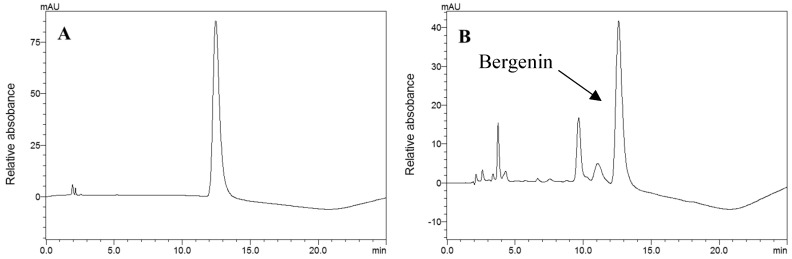
HPLC profile of the bergenin standard (**A**) and the aqueous bark *Endopleura uchi* extract (**B**) analyzed at 272 nm.

The bark extract obtained from *E. uchi* showed an antioxidant capacity in vitro as powerful as standard dietary antioxidants, such as vitamin C and EGCG, when tested in DPPH assay (Table 1). Correspondingly, a high content of phenolics was observed using Folin-Ciocalteu method (850 GAE/g extract). Through HPLC, the isocoumeric bergenin was found to be the major compound in the extract (4.5 g/100 g of dry extract; Figure 1).

### 3.2. Effect of the Extract on Intracellular ROS Accumulation

**Figure 2:**
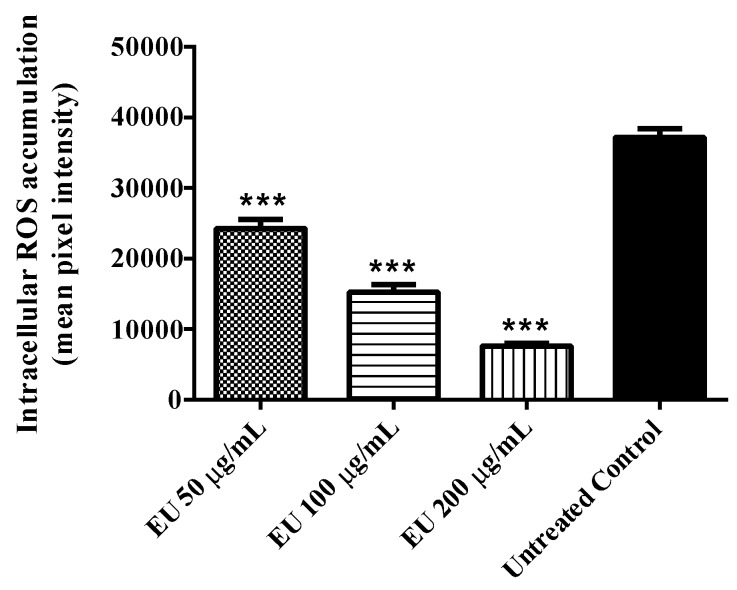
Quantification of intracellular ROS in N2 worms using DCFDA after treatment with *Endopleura uchi* extract (EU). Worms treated with EU showed lower levels of ROS compared to the control group. Data are presented as mean pixel intensity ± SEM (*n* = 40, replicated 3 times). *** *p* < 0.001, compared to the untreated control by one-way ANOVA followed by Bonferroni (*post-hoc*).

Endogenous intracellular ROS production was investigated in wild type (N2 (wt)) worms under stress-free conditions. The result obtained indicated a significant decrease in ROS accumulation among worms treated with the EU as compared with the untreated control group. The decrease was up to 80% when the worms were treated with 200 µg/mL EU (adjusted *p*-value < 0.0001; Figure 2).

### 3.3. Protection against Oxidative Stress

**Figure 3:**
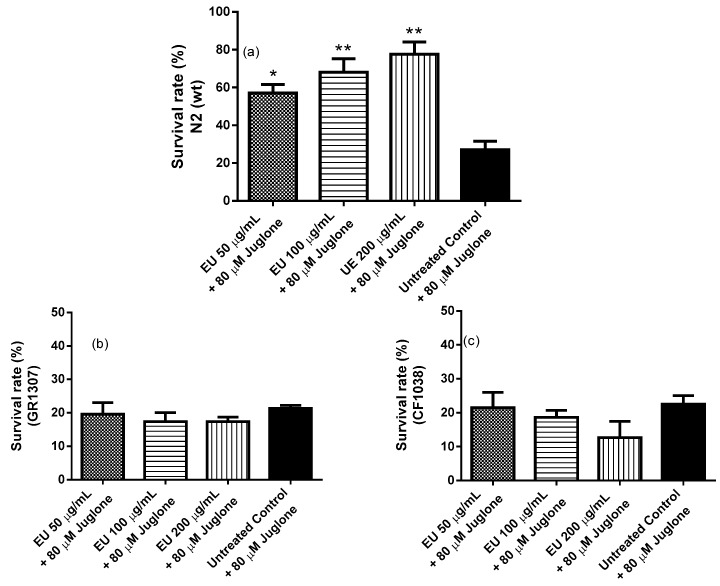
Survival of *C. elegans* after juglone-induced oxidative stress. Survival rate of N2 worms was significantly enhanced in the groups treated with the bark extract (EU) (**a**). However, the survival rate of DAF-16 mutants (GR1307 [*daf-16*(mgDf50) I] and CF1038 [*daf-16*(mu86) I]) did not differ between the groups (**b**) and (**c**), respectively. Each bar represents the mean ± SEM from three independent assays. Note: * *p* < 0.05 and ** *p* < 0.01 compared to the untreated control by one-way ANOVA followed by Bonferroni’s method (*post-hoc*).

Protection of the worms against oxidative stress by the extract was assessed by comparing the survival rate of wild type worms (N2 (wt)) after juglone-induced oxidative stress. The results indicated a significant higher survival rate among EU treated worms. At the highest tested concentration (200 µg/mL EU), 77% of the worms remained alive after juglone exposure compared to 27% scored in the extract-free group (adjusted *p*-value = 0.0017; Figure 3a).

In order to find out if DAF16-FOXO pathway plays a role in the stress resistance observed after EU treatment, we performed the same protocol using mutant strains, in which DAF16 was inactivated (strains: CF1038 (*daf-16*(mu86)I) and GR1307 (*daf-16*(mgDf50)). As illustrated in Figure 3b,c, these mutants did not benefit from the antioxidant properties of EU as did the wild type worms (N2 (wt)).

### 3.4. Effect of the Extract on the Expression of Stress Response Genes (hsp-16.2::GFP and sod-3::GFP)

**Figure 4:**
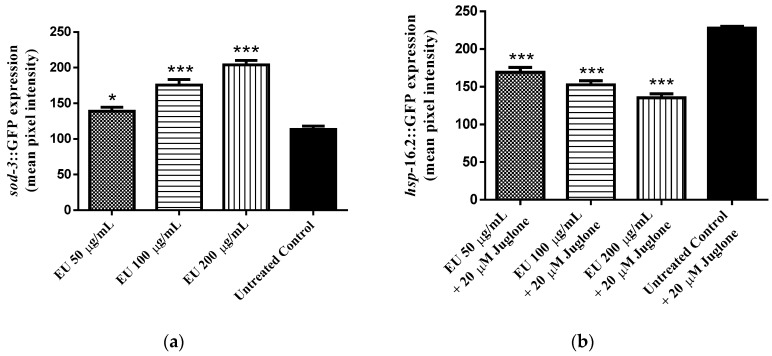
Expression of stress response genes. CF1553 worms [(pAD76)*sod-3*p::GFP + rol-6] treated with *Endopleura uchi* (EU) showed significant higher levels of SOD-3::GFP compared to the untreated control group (**a**), and mutant worms TJ375 [*hsp-16.2*::GFP(gplsI)] exposed to 20 µM juglone presented significant lower levels of HSP-16.2::GFP when compared with the untreated control worms similarly exposed to 20 µM juglone (**b**). Data are presented as mean pixel intensity (mean ± SEM) from three independent experiments. Note: * *p* < 0.05 and *** *p* < 0.001 related to the control, analyzed by one-way ANOVA followed by Bonferroni (*post-hoc*).

The expression of *sod-3* was investigated using mutant worms (strain CF1553), in which *sod-3* has been fused with a GFP reporter. From analyses of the emitted fluorescence, we observed a significant increase in *sod-3*::GFP expression by 44% among EU treated worms compared with the untreated control group (adjusted *p*-value < 0.0001; Figure 4a).

The expression of *hsp*-*16.2* was assessed using the mutant strain TJ375, in which *hsp-16.2* is fused with GFP. After mild oxidative stress, induced by adding a low concentration of juglone to the medium, we observed a significant fluorescence in the nematodes. The intensity was reduced among EU treated worms as compared with untreated worms. The decrease was up 40% at a concentration of 200 µg/mL EU (adjusted *p*-value < 0.0001; Figure 4b).

### 3.5. Effect of the Extract on Longevity

**Figure 5:**
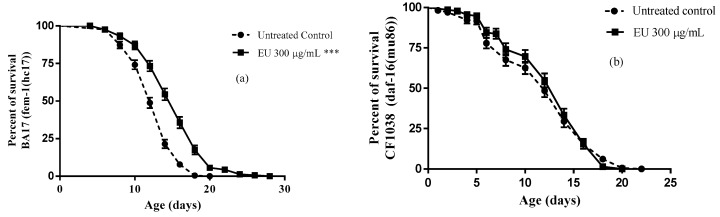
Longevity of *C. elegans* after treatment with *Endopleura uchi* extract (EU). BA17 worms treated with EU 300 µg/mL presented significantly longer lifespan compared to untreated control group (**a**). However, lifespan of *daf-16* null mutants (CF1038) were no significantly different between EU treated and untreated worms (**b**). The results are presented as percentage of surviving worms and the statistical significance determined by Log-rank (Mantel-Cox) tests followed by Gehan-Breslow-Wilcoxon Test. Note: *** *p* < 0.001.

A long-term assay was performed to test whether EU can influence longevity in *C. elegans*. The results obtained indicated extension of lifespan by 33% among BA17 worms treated with EU as compared to untreated control group (*p*-value *p* < 0.0001; Figure 5a). However, when the assay was performed with the *daf*-16 null mutants (CF1038 strain) no significant difference in the mean lifespan was observed between treated and untreated worms (Figure 5b), indicating that the transcription factor DAF16 plays a role in this context.

### 3.6. Effect of the Extract on the Pharyngeal Pumping Rate

**Figure 6:**
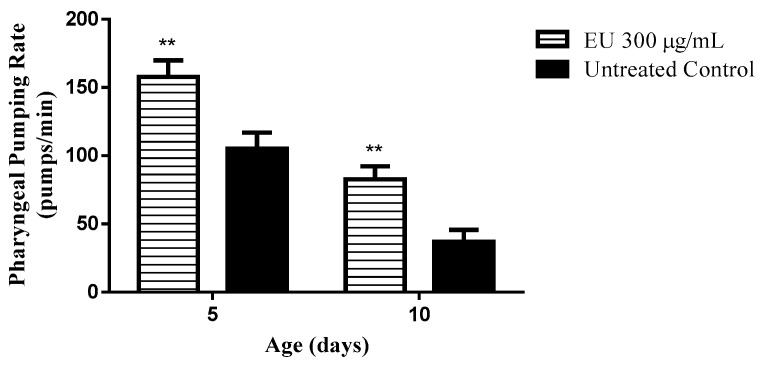
Pharyngeal pumping rate in *C. elegans* after treatment with the bark extract (EU). The treatment of wild type worms with EU 300 μg/mL significantly attenuated the age-associated decline in the muscle function of pharynx. Data are presented as mean ± SEM. Note: ** *p* < 0.01 related to the control by a two-way ANOVA.

During aging, muscle activity is impaired. As a marker for muscle activity, the pharyngeal pumping activity can be monitored in *C. elegans*. The pumping activity of the pharynx was scored in wild type (N2 (wt)) worms at day 5 and 10 of adulthood and revealed a significant difference among the groups. Worms cultured in medium supplemented with EU exhibited an improved pumping function of the pharynx. At day 10, the pumping rate among EU treated worms was 128% higher than that scored among untreated worms (*p*-value < 0.01; Figure 6). The data indicates that the muscle function is better preserved in worms under EU treatment as they age. Such a result also indicates that EU treated worms did not starve during their lifetime, so caloric restriction effect can be ruled out.

### 3.7. Body Length

Body length is an important measurement to evaluate the possible deleterious effect of DR in *C. elegans*. In the current study, the body length of adult wild type (N2 (wt)) worms was compared between those who had been under EU treatment and the untreated ones. The data obtained indicated no differences between the groups. The treatment group had a length of 1.339 ± 0.018 mm as compared to controls with of 1.261 ± 0.018 mm. The result indicates that the worms did not undergo caloric restriction or any toxic effect able to impair body development while treated with EU extract.

### 3.8. Antimicrobial Activity

The bark extract was tested against *E. coli* OP50, bacterial strains used to feed *C. elegans*, and no bactericidal effect from EU was observed (Table 2). This finding further indicates that the worms did not undergo caloric restriction during the treatment due to a reduction of food source.

### 3.9. Effect of the Bark Extract on the Formation of polyQ40 Aggregates

**Figure 7:**
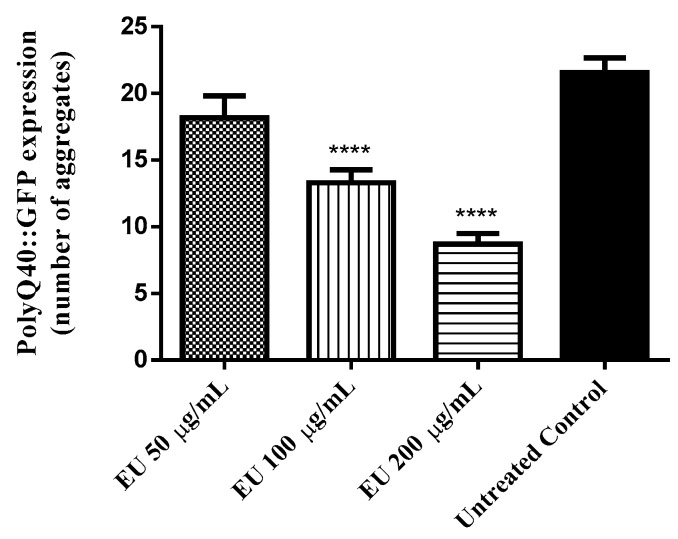
PolyQ40::GFP aggregate formation in mutant worms (AM141) after treatment with *Endopleura uchi* extract (EU). Worms treated with EU exhibited significant lower number of polyQ40::GFP aggregates compared to the control group. Data are presented as mean ± SEM. Note: *** *p* < 0.001 related to the control by a one-way ANOVA followed by Bonferroni (*post-hoc*).

In Huntington’s disease, the disease-causing Huntington gene is mutated and carries many glutamate repeats. The formation of polyQ40 aggregates was assessed in AM141 mutants, which produce polyQ fused with GFP. The results obtained indicated a significant lower number of polyQ40::GFP aggregates among EU treated worms. At the highest tested concentration, 300 µg/mL EU, the number of fluorescent aggregates scored was reduced by 60% when compared with the untreated control group (adjusted *p*-value < 0.0001; Figure 7).

## 4. Discussion

The Folin-Ciocalteu assay indicated a high phenolic content in EU water extract. HPLC UV/VIS analyses of our extract identified the phenolic bergenin as a major component, in line with the literature [17,19]. Bergenin has been reported as an antioxidant, anti-HIV, gastroprotective, neuroprotective, hepatoprotective, and immunomodulatory agent [25,26,27,28,29], effects that could explain the traditional uses of the barks of uxi by the locals in Amazonia.

The high antioxidant activity in vitro was also observed in vivo using *C. elegans* as a model organism. Notably, wild type worms treated with EU exhibited a higher survival rate after induced oxidative stress as compared to untreated worms submitted to identical conditions. These data demonstrate the capacity of the extract to counteract oxidative damage promoted by exogenous sources such as the pro-oxidant juglone [30]. In agreement, in EU treated worms we found lower accumulation of endogenous cellular ROS and lower pattern of expression for *hsp*-16.2, the gene that codes for HSP-16.2, a small heat shock protein whose expression is induced in response to harsh cellular conditions such as heat stress and oxidative damage [31,32].

Polyphenolic rich extracts are proposed to enhance cellular stress resistance through modulation of stress response genes in addition to free radical scavenging activities [33]. In the current study, *sod-3*, the gene coding for the mitochondrial antioxidant enzyme superoxide dismutase 3, showed higher expression among worms treated with EU. The upregulation of *sod-*3 suggests the participation of the transcription factor DAF-16, the *C. elegans* orthologue for the mammalian FOXO transcription factor, whose target genes are mainly involved in stress resistance, metabolism, and longevity [34,35]. When testing EU in DAF-16 null mutants (CF1038 and GR1307 strains), we noticed that the protecting effect of the extract, previously demonstrated in wild type worms submitted to the survival assay, was absent. The data, therefore, confirms the requirement of DAF-16 transcription factor to promote the antioxidant effect of EU.

Plant extracts with a high content of polyphenolic compounds, such as those obtained from *Camellia sinensis*, *Calycophyllum spruceanum*, and *Paullinia cupana*, have been shown to extend lifespan in *C. elegans* due to their capacity to modulate molecular mechanisms that drive cellular stress resistance and metabolism, in line with the free radical theory of aging [22,23,36]. Considering the pronounced in vivo antioxidant activity of EU elicited by its capacity to modulate stress response genes in DAF-16 pathway, we also investigated whether this extract could affect longevity in *C. elegans* and have found a positive result. The mean lifespan of the EU treated worms was increased by 33%, an effect that was absent in DAF-16 null mutants, indicating a molecular basis underlying it.

However, lifespan extension is not essentially followed by an extension of healthspan, the period of life free from diseases. The fundamental mechanisms underlying both are distinct and complex. Authors highlight that the sole extension of lifespan might not be desirable if it represents just an extended period of frailty, where individuals are vulnerable to aging-related diseases [37,38].

In literature, the role of oxidative stress on the onset of aging-related diseases is well documented, thus several antioxidant compounds are claimed to be capable of attenuating or preventing the impact of aging [39]. In agreement, we obtained evidence for an anti-aging effect of EU in *C. elegans* by studying its muscle function, which works as marker of aging [40,41]. Analyzing the results, we observed a higher contraction rate among worms treated with the extract at all scored timepoints. This data indicates that the treatment can attenuate the age-related muscle function decline, which is considered one important aspect of the healthspan to be maintained to achieve the so-called successful aging [42].

Caloric restriction is a well-known pro-longevity stimulus [43]. To investigate whether the worms could have faced caloric restriction during the period when they were under treatment, we tested the extract against *E. coli* OP50 and did not find a bactericidal effect. Moreover, the pharyngeal contractile capacity of the treated worms was higher as compared to untreated worms, and when we measured the body length we found no differences between treated and untreated worms. The data indicate that the worms did not starve at any timepoint of their lifespan. Thus, we assume that EU can effectively extend lifespan in *C. elegans* by molecular mechanisms other than caloric restriction.

Neurodegenerative diseases are more prevalent in elderly and dramatically impair life quality. In this context, we decided to treat mutant worms expressing polyQ40, an expanded series of glutamine residues involved in the pathophysiology of Huntington’s disease [44,45]. Our data indicate that EU treatment is able to attenuate the formation of polyQ40 aggregates, another result supporting the anti-aging properties of EU which needs to be studied in more detail.

In conclusion, the polyphenol-rich water extract from the stem bark of *Endopleura uchi* exhibited substantial antioxidant activity in vitro and in vivo. The extract was able to enhance the stress resistance in *C. elegans* through the modulation of the DAF-16/FOXO pathway. Additionally, the extract exhibited anti-aging properties being able to extend lifespan and to attenuate markers of aging, such as age-related muscle function decline and the formation of polyQ40 aggregates. Considering the traditional application of the bark of uxi and its extensive use by the local population, more studies are needed to elucidate the biological activities as well as its toxicological profile in detail.

## Figures and Tables

**Table 1:** Antioxidant activity of *Endopleura uchi* (EU) assessed by DPPH assay.

SAMPLE	EC50 (µG/ML)
EU	8.0
EGCG	1.2
VITAMIN C	2.1

**Table 2:** Antimicrobial activity of the *Endopleura uchi* (EU) bark extract assessed by well diffusion test.

	Ampicillin (256 µg/mL)	Ciprofloxacin (256 µg/mL)	EU (10 mg/mL)
***E. coli* OP50**	27.3 ± 1.2 *	42.8 ± 0.3 *	NI

* Zone of inhibition (mm); NI: no inhibition.
